# Supplementary material for: Efficient protein targeting to the inner nuclear membrane requires Atlastin-dependent maintenance of ER topology
Source: eLife. 2017 Aug 14;6:e28202. doi: 10.7554/eLife.28202 (PMC5587084; doi:10.7554/eLife.28202)
Supplement: Transparent reporting form [file elife-28202-transrepform.docx]

***eLife’s* transparent reporting form**

We encourage authors to provide detailed information *within their submission* to facilitate the interpretation and replication of experiments. If you have any questions, please contact us: [editorial@elifesciences.org](mailto:editorial@elifesciences.org).

**Sample-size estimation**

- You should state whether an appropriate sample size was computed when the study was being designed
- You should state the statistical method of sample size computation and any required assumptions
- If no explicit power analysis was used, you should describe how you decided what sample (replicate) size (number) to use

Please outline where this information can be found within the submission (e.g., page numbers or figure legends), or explain why this information doesn’t apply to your submission:

No explicit power analysis was used to determine the sample size for each experiment. Sample size was based on empiric considerations taking into account both robustness of the respective assay and the experimental challenge of acquiring the data. Usually the sample size was high for in vitro assays (e.g. in vitro INM targeting assay) or assays performed on fixed cells (e.g. immunofluorescence) and lower for assays performed using living cells.

**Replicates**

- You should report how often each experiment was performed
- You should include a definition of biological versus technical replication
- The data obtained should be provided and sufficient information should be provided to indicate the number of independent biological and/or technical replicates
- If you encountered any outliers, you should describe how these were handled
- Criteria for exclusion/inclusion of data should be clearly stated
- High-throughput sequence data should be uploaded before submission, with a private link for reviewers provided (these are available from both GEO and ArrayExpress)

Please outline where this information can be found within the submission (e.g., page numbers or figure legends), or explain why this information doesn’t apply to your submission:

All experiments represented in the primary figures were performed at least in triplicates except for Figs. 1, 2B and 6C. The number of times each experiment has been performed is indicated as ‘N’ in figure legends. These refer to the number of times the entire experiment was performed on independent days and therefore correspond to biological replicates of the experiment. Detailed description of the number of cells analyzed per data set can be found in the figure legends, represented by ‘n’.

**Statistical reporting**

- Statistical analysis methods should be described and justified
- Raw data should be presented in figures whenever informative to do so (typically when N per group is less than 10)
- For each experiment, you should identify the statistical tests used, exact values of N, definitions of center, methods of multiple test correction, and dispersion and precision measures (e.g., mean, median, SD, SEM, confidence intervals; and, for the major substantive results, a measure of effect size (e.g., Pearson's r, Cohen's d)
- Report exact p-values wherever possible alongside the summary statistics and 95% confidence intervals. These should be reported for all key questions and not only when the p-value is less than 0.05.

As indicated in the materials and methods section, we have performed Student’s t-test to calculate the statistical significance of our data. P-values were calculated using Prism and have been reported in the corresponding figure legends. The number of replicates, number of cells, and precision measures (SD or SEM) can also be found in the figure legends.

Please outline where this information can be found within the submission (e.g., page numbers or figure legends), or explain why this information doesn’t apply to your submission:

(For large datasets, or papers with a very large number of statistical tests, you may upload a single table file with tests, Ns, etc., with reference to page numbers in the manuscript.)

**Additional data files (“source data”)**

- We encourage you to upload relevant additional data files, such as numerical data that are represented as a graph in a figure, or as a summary table
- Where provided, these should be in the most useful format, and they can be uploaded as “Source data” files linked to a main figure or table
- Include model definition files including the full list of parameters used
- Include code used for data analysis (e.g., R, MatLab)
- Avoid stating that data files are “available upon request”

Please indicate the figures or tables for which source data files have been provided:

Manuscript does not contain source data
